# CAR T cell cancer immunotherapy: who does the job?

**DOI:** 10.3389/fimmu.2026.1955025

**Published:** 2026-09-16

**Authors:** Dennis Christoph Harrer, Markus Barden, Hinrich Abken

**Affiliations:** 1 Leibniz Institute for Immunotherapy, Div. Genetic Immunotherapy, Regensburg, and Chair Genetic Immunotherapy, University Regensburg, Regensburg, Germany; 2 Department of Internal Medicine III, Hematology and Oncology, University Hospital Regensburg, Regensburg, Germany

**Keywords:** anti-tumor response, CAR - T therapy, chimeric antigen receptor (CAR), innate cell, T cell

## Abstract

Chimeric antigen receptor (CAR) T cells are capable to eliminate cancer cells in the treatment of hematologic malignancies, yet the efficacy is frequently inconsistent and limited by cancer cell resistance and antigen-loss resulting in early tumor relapses. While CAR T cells are deemed to be the primary effectors in controlling the tumor, maturing evidence indicates that therapeutic outcomes are shaped by a broader immune cell network involving both the endogenous adaptive and innate immunity. In this review, we reframe CAR T cell therapy as the induction of a multi-system immune response rather than a uni-directional cytotoxic cell-autonomous intervention. We discuss the respective contributions of CAR T cells, innate immune cells, and host adaptive immunity in controlling tumor progression and outline strategies to recruit innate immunity using TRUCKs, armored CAR T cells, as well as immunological adjuvants to surmount current limitations. Finally, we address the risks associated with excessive innate immune activation and propose that a calibrated, broad immune cell activation should be viewed as design principle for next-generation CAR T cell therapies.

## Introduction

Chimeric antigen receptor (CAR) T cells represent a breakthrough in the immunotherapy of cancer, particularly in B cell malignancies (1, 2). By redirecting patient-derived T cells to recognize tumor-associated antigens independently of major histocompatibility complex (MHC) restriction, CAR T cells can destruct malignant cells upon target engagement and ensuing cytolytic activation (3, 4). Clinical responses in the treatment of refractory leukemias and lymphomas have demonstrated the transformative potential of the approach (1, 2). However, success is tempered by substantial variability in patient outcomes and limited efficacy in the treatment of solid tumors (5, 6). The majority of patients exhibits primary resistance, often due to inadequate CAR T cell expansion, T cell exhaustion and poor persistence, or an immunosuppressive tumor microenvironment (TME) (5, 7). Various factors, including low antigen density or heterogeneous expression, further compromise the therapeutic efficacy (8). Even in responders, a multitude of patients experience disease recurrence after transient remissions, reflecting failure in establishing a long-term immune surveillance (9–11). In addition, the TME imposes multiple barriers, including physical fibrotic walls, metabolic constraints, hypoxia, and the activities of suppressive immune cell populations (5, 6, 12). Finally, loss of the CAR cognate antigen and clonal cancer cell selection, also known as “antigen escape”, favors tumor relapses (13, 14).

To address these challenges, it is increasingly important to move beyond the current CAR T cell paradigm of focusing exclusively on the direct cytotoxic activity of engineered T cells as the primary effector mechanism, but to contemplate the broader immunological context in which CAR T cell therapy operates. The notion that CAR T cells do not act in isolation but rather can be used to initiate a cascade of immune events that engage both innate and adaptive immune compartments has gained mounting recognition over recent years (15–21). Scant clinical evidence supports a tight interconnection between innate and adaptive immunity to control tumor. Case in point: Two ALL cases achieved sustained remissions after severe fungal infections, i.e., mucormycosis and aspergillosis, respectively, that forced cessation of chemotherapy, however, accompanied by tumor remission that maintained under antimicrobial therapy alone. This observation, although anecdotical, support the idea that strong infections may trigger innate immune activation, potentially via Toll-like receptor (TLR) sensing of pathogen-associated molecular patterns (PAMPs), shifting immunity toward an anti-leukemic state and contributing to tumor remission (22). The understanding how different immune cell populations contribute to and maintain tumor clearance may provide a conceptual framework for improving clinical outcomes of CAR T cell therapy (15, 19).

The evolving notion raises a central question: Who does the job in CAR T cell therapy? By integrating CAR T cells with innate and adaptive immune responses, we will dissect their contributions and explore strategies to actively combine CAR T cells with innate immune cell engagement, leveraging engineered CAR T cell platforms such as T cells redirected for universal cytokine killing (TRUCKs) and armored CAR T cells, as well as the use of immunological adjuvants or vaccines. Finally, we will also illuminate the potential risks associated with excessive innate immune activation, including cytokine release syndrome (CRS), macrophage activation syndrome (MAS) and hemophagocytic lymphohistiocytosis (HLH). Taking the emerging developments together we will draw a vision how to achieve a calibrated activation of innate immunity triggered by CAR T cells to optimize the therapeutic benefit while minimizing toxicities.

### CAR T cell cancer immunotherapy: are CAR T cells the only drivers?

CAR T cells mediate direct and antigen-specific cytotoxicity against cancer cells through recognition of surface antigens, such as CD19, carcinoembryonic antigen (CEA), or others (23, 24). Upon engagement with their target, CAR T cells become activated and deploy canonical effector mechanisms, including perforin- and granzyme-mediated lysis, Fas/FasL interactions, and the secretion of pro-inflammatory cytokines such as IFN-γ, TNF-α, and TRAIL (25). In addition to immediate cancer cell killing, CAR T cells proliferate extensively *in vivo* and can differentiate into memory-like populations, supporting sustained immune surveillance (26). However, T cell exhaustion, triggered by chronic antigen exposure and inhibitory signaling within the TME, poses a prime impediment limiting CAR T cell persistence and efficacy (5, 7, 27). As a result, the CAR T cell antitumor response as standalone activity is likely insufficient to induce complete and lasting remissions in most patients.

While CAR T cells are engineered as the primary effectors of cancer cell killing, their therapeutic activity to eradicate tumors is embedded in a concerted, multi-layered immune response involving both innate and endogenous adaptive immunity. Innate immune cells, notably macrophages, dendritic cells, and natural killer (NK) cells, assume a critical but often underappreciated role in CAR T cell therapy by their capacity to amplify tumor reduction (15, 16, 20, 28, 29). Robust cytokine release along with CAR T cell activation in turn recruits and activates myeloid cells, preferentially within the TME where CAR T cell activation occurs (30, 31). Case in point, macrophages activated via IFN-γ can phagocytose cancer cells and produce additional inflammatory mediators such as IL-1 and IL-6, thereby reinforcing local immune activation (32). In addition, released antigens ensuing cancer cell lysis by CAR T cells are processed and presented by dendritic cells to “bystander” T cells, thereby bridging innate and adaptive immunity (17, 33). Moreover, NK cells can basically contribute through antigen-independent cytotoxicity, particularly against cancer cells that lost MHC expression (34).

Tumor cell destruction by CAR T cells may lead to the release of a broad repertoire of tumor antigens, which can be taken up by antigen-presenting cells and cross-presented to host T cells. This process can trigger “epitope spreading,” whereby non-CAR T cell responses are broadening the antitumor immune response by targeting additional tumor antigens (17, 20, 28). Conceivably, endogenous cytolytic CD8^+^ T cells, primed in the draining lymph node, can thereby contribute to sustained tumor control, particularly in settings where antigen escape would otherwise lead to relapse. On top of that, CD4^+^ T cells could further support the response by providing cytokine help coordinating the immune network.

Collectively, these observations support a model in which CAR T cells may primarily act as initiators of a broader immune cascade rather than as solitary effectors (**Figure 1**). Their ability to directly lyse tumor cells would be necessary but, for its own, frequently proves insufficient to cure cancer patients. Instead, optimal therapeutic outcomes would likely depend on a productive crosstalk with innate immune cells and the successful priming of the endogenous adaptive immunity. Dissecting the relative contributions of these components is essential for the understanding of treatment success, resistance, and toxicity. The integrated perspective provides the basis for next-generation strategies aimed at harnessing and fine-tuning these interactions. Recent clinical evidence further supports a role for endogenous immunity in CAR T-cell efficacy. In recurrent glioblastoma, CAR T-cell activation was observed in both responders and non-responders, with CAR T-cell-intrinsic features not clearly distinguishing outcomes. Responders, however, showed expansion of cytotoxic NK cells, whereas non-responders exhibited increased regulatory T cells and immunosuppressive myeloid cells (35). Similarly, in a comprehensive analysis of 256 patients across 13 CAR T-cell trials, early serum IL-18 and IL-6 levels at day 7 distinguished responders from non-responders, suggesting that early innate immune activation may contribute to effective CAR T-cell responses (30).

**Figure 1:**
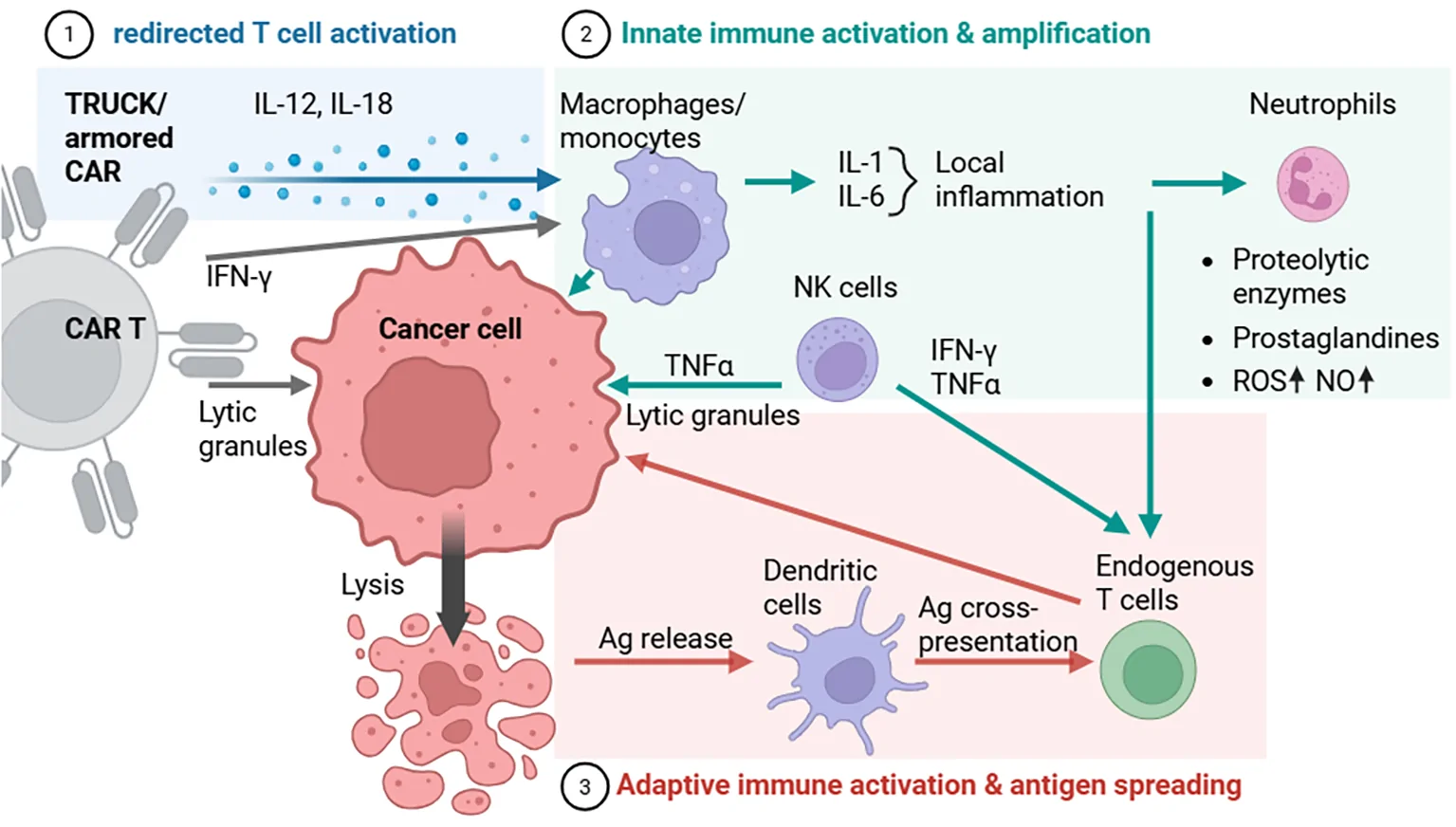
The multi-effector cascade. Who does the job?. Created with BioRender.

## Combined CAR T cell immunotherapy: merits of recruiting innate immunity

Given the growing recognition that CAR T cells act within an immunological network, strategies to actively recruit innate immunity have attracted considerable attention. Engaging innate immune components could basically amplify the antitumor efficacy through multiple mechanisms, including enhanced local inflammation, improved antigen presentation, and promotion of endogenous adaptive immune responses. Overall, the concomitant activation of an innate immune response could help surmount some limitations of CAR T cell therapy, such as the poor activity in the immunosuppressive TMEs, limited persistence, and antigen escape. Accordingly, next-generation CAR T cell approaches are aiming to convert tumors into immunologically “hot” environments by coupling CAR T cell cytotoxicity with a localized activation of innate immune cells (6, 36, 37).

One type of modulating the immune cell environment are TRUCKs that constitute the “fourth generation” of CAR T cells engineered to deliver a transgenic cytokine or other payloads upon antigen engagement in a locally restricted fashion (38). These constructs incorporate an inducible expression system for transgenic proteins, commonly for cytokines such as IL-12 (15), IL-18 (16), or type-I interferons, that are produced and released downstream of CAR signaling (39). Upon encountering cancer cells, TRUCKs secreting IL-12 or IL-18 not only augment direct T cell mediated cytotoxicity but also recruit and activate innate immune cells through the released cytokines, including macrophages and NK cells (15, 16). As a result, the antigen presentation by dendritic cells is enhanced promoting a pro-inflammatory milieu that further supports a broader immune activation and infiltration by additional CAR T cells (16). Strikingly, TRUCKs can also help to bypass antigen restriction by enabling antigen-independent killing through activated innate immune effectors, thereby mitigating the risk of tumor resistance due to antigen heterogeneity and loss (15).

Armored CAR T cells are engineered to resist immunosuppression and actively modulate the TME through constitutive expression of additional functional elements beyond the CAR. These modifications include secretion of pro-inflammatory cytokines like IL-18 (40, 41), expression of dominant-negative receptors to block TGF-β signaling (42), or ligands that stimulate innate immune cells, such as CD40L (20, 28), or TLR-4 (43). By enhancing resistance to inhibitory signals and simultaneously activating bystander immune cell populations, armored CAR T cells can locally sustain their effector function in the otherwise hostile environment. Engaging innate immune pathways improves the recruitment and activation of antigen-presenting cells, thereby strengthening the link between innate and adaptive immunity. The integrated approach not only enhances direct cancer cell killing but also supports the development of a more durable and diversified immune response.

In addition to cell-intrinsic engineering strategies, pharmacological adjuvants offer an actionable approach to boost innate cell engagement during CAR T cell therapy. Agents such as TLR agonists (44), STING agonists (45), mRNA adjuvants (9, 21, 46, 47), or oncolytic viruses can be used to stimulate innate immune pathways within the tumor (48). These adjuvants promote the production of type-I interferons and other inflammatory mediators, enhancing dendritic cell activation and antigen presentation. As a result, epitope spreading and endogenous T cell responses are augmented and the TME reshaped by reducing suppressive cell populations and improving immune cell infiltration. When appropriately timed and dosed, these approaches have the potential to enhance the breadth and durability of antitumor immunity.

Collectively, strategies that recruit innate immunity transform CAR T cell therapy from a purely targeted cytotoxic intervention into a more comprehensive immunological assault on the tumor. By leveraging the innate immune system and its capacity in orchestrating downstream adaptive responses, these approaches hold considerable promise for resolving current therapeutic limitations.

## Caveats in recruiting innate immunity

While the recruitment of innate immunity can substantially enhance the efficacy of CAR T cell therapy, it also harbors significant risks that originate from excessive or dysregulated immune activation. The same inflammatory pathways can promote tumor clearance and, when uncontrolled, may stir severe systemic toxicities (49). In this context, innate immune cells, particularly monocytes and macrophages, are not merely supportive effectors but can also act as central drivers of inflammatory severe adverse events (49). As strategies such as TRUCKs, armored CAR T cells, and immunostimulatory adjuvants are aiming to increasingly amplify innate cell engagement, careful consideration of these risks becomes imperative.

Cytokine release syndrome is the most prevalent significant toxicity associated with CAR T cell therapy and is closely linked to the activation of innate immune pathways (50). Although CAR T cells initiate the response through antigen recognition and cytokine secretion, such as IFN-γ and TNFα, the predominant hypothesis indicates that myeloid cells, especially macrophages, are the key amplifiers of the cytokine cascade (49). Activated macrophages produce high levels of IL-6, IL-1, and other pro-inflammatory mediators, leading to a systemic hyperinflammatory state. Clinically, CRS can range from mild flu-like symptoms to life-threatening manifestations, including high fever, hypotension, vascular leakage, and multi-organ dysfunction (51, 52). Importantly, the severity of CRS often correlates with tumor burden and the magnitude of immune activation, highlighting the fine balance between therapeutic efficacy and toxicity (53). Therapeutic interventions such as IL-6 receptor blockade with tocilizumab have improved management, but do not fully abrogate the complexity of the syndrome, particularly when multiple inflammatory pathways are engaged (51).

A more severe and less common complication is macrophage activation syndrome, also referred to as immune effector cell-associated HLH-like syndrome (IEC-HS) in the context of CAR T cell therapy (54). This condition represents an extreme form of immune dysregulation characterized by uncontrolled activation and expansion of macrophages and cytotoxic lymphocytes (55). Similar to CRS, IEC-HS is driven by excessive cytokine production, including high levels of IL-6, IL-1, IFN-γ, and ferritin, but is distinguished by additional features such as cytopenias, coagulopathy, liver dysfunction, and hemophagocytosis (55). The pathophysiology reflects a failure of immune homeostasis, in which persistent activation signals and disturbed negative feedback mechanisms lead to a self-amplifying inflammatory loop. In theory, therapeutic strategies that intentionally boost innate immune activation, such as IL-12 or IL-18 secreting TRUCKs (56) or strong pattern recognition receptor agonists, increase the risk of triggering such hyperinflammatory states if not tightly controlled. Notably, first clinical evidence from CAR T cells constitutively secreting IL-18 do not indicate major toxicity beyond that typically associated with CAR T cell therapy (40).

Collectively, these toxicities underscore a fundamental tightrope challenge in next-generation CAR T cell design: the need to harness the beneficial aspects of innate immune activation in a localized, well-tuned fashion without tipping the balance toward systemic immunopathology. While innate immunity can significantly potentiate antitumor responses, its powerful and rapid amplification capacity renders it inherently difficult to regulate once fully engaged. Therefore, understanding the cellular and molecular drivers of CRS and IEC-HS is critical for developing both safer and more efficacious therapeutic strategies that achieve a broad, yet controlled, immune activation.

## Calibrated activation of innate immunity to boost CAR T cell cancer immunotherapy

CAR T cells are essential initiators of tumor targeting and lysis, but their therapeutic effect is best understood as part of a coordinated immune response involving both innate and endogenous adaptive immunity. Rather than acting as isolated effectors, CAR T cells function as orchestrators that shape a broader immune cascade leading to tumor eradication (**Figure 2**). Future therapeutic strategies are therefore likely to focus less on maximizing intrinsic CAR T cytotoxicity and more on coordinating local immune networks within the tumor microenvironment. Key opportunities include enhancing antigen presentation and engaging bystander T cells and innate immune cells, particularly through engineered approaches such as TRUCKs and armored CAR T cells. CAR T cells secreting IL-18 exemplify this concept by recruiting and activating multiple immune populations to amplify antitumor activity. However, such immune amplification must be carefully balanced against the risk of excessive inflammation and toxicity.

**Figure 2:**
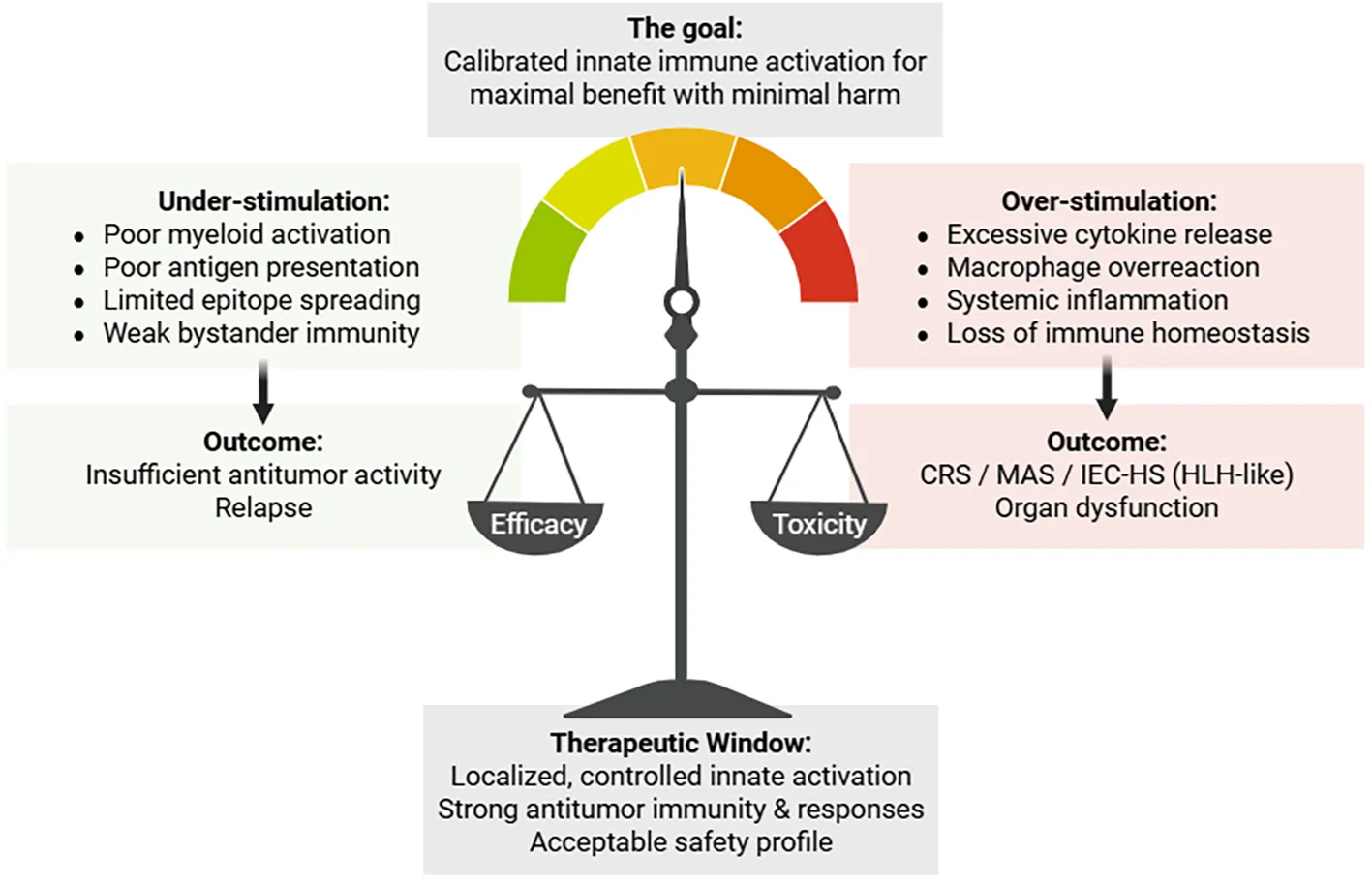
The efficacy-toxicity calibration spectrum. Created with BioRender.
